# OPTIMAL AGING AND SATISFACTION WITH SOCIAL SUPPORT DURING THE COVID-19 PANDEMIC

**DOI:** 10.1093/geroni/igac059.1949

**Published:** 2022-12-20

**Authors:** Hye Soo Lee, Soyoung Choun, Maria Kurth, Heidi Igarashi, Dylan Lee, Carolyn Aldwin

**Affiliations:** Oregon State University, Corvallis, Oregon, United States; Oregon State University, Corvallis, Oregon, United States; Oregon State University, Corvallis, Oregon, United States; Oregon State University, Corvallis, Oregon, United States; Touro University Nevada, Henderson, Nevada, United States; Oregon State University, Corvallis, Oregon, United States

## Abstract

Social support is important for optimal aging, especially during the COVID-19 pandemic when older adults are at risk of social isolation and its attendant health problems. Providing support may be especially protective of health outcomes. We examined whether actual received, provided, and satisfaction with support were related to optimal aging early in the pandemic (April-May, 2020). Survey participants (N=238) were on average 71.2 years old (SD=7.3), 73% female, 92.6% White, and highly educated (48% with post-graduate degrees). Optimal aging (Aldwin & Igarashi, 2016) was indicated by a latent variable of health outcomes, including depressive symptoms, cognitive lapses, and physical symptoms. Nearly all older adults (90+%) reported receiving or providing actual support from or to at least one family member or friend. We investigated the associations between age, summed social support, satisfaction with support, and health outcomes, controlling for chronic illnesses. Two SEM models were estimated for received support and provided support, respectively. After trimming non-significant paths, both models had acceptable fits (CFI > .90, RMSEA < .08, SRMR < .08). Age and chronic illnesses had negative associations with health outcomes, but neither received nor provided social support was significant. However, satisfaction with both received and provided support were significant and independently associated with optimal aging in both models. Thus, isolation levels in this sample were surprisingly low, as indicated by high levels of social support received and provided. However, only the quality of (or satisfaction with) support was important for optimal aging during this unique and shared stressful experience.

